# Harvesting Variable-Speed Wind Energy with a Dynamic Multi-Stable Configuration

**DOI:** 10.3390/ma13061389

**Published:** 2020-03-19

**Authors:** Yuansheng Wang, Zhiyong Zhou, Qi Liu, Weiyang Qin, Pei Zhu

**Affiliations:** 1 Department of Engineering Mechanics, Northwestern Polytechnical University, Xi’an 710072, China; wangyuansheng@nwpu.edu.cn (Y.W.); 10160091@vip.henu.edu.cn (Z.Z.); liuqiarc@163.com (Q.L.); 10160096@vip.henu.edu.cn (P.Z.); 2 School of Civil Engineering and Architecture, Henan University, Kaifeng 475004, China

**Keywords:** wind energy harvesting, snap-through motion, dynamic stability, variable-speed

## Abstract

To harvest the energy of variable-speed wind, we proposed a dynamic multi-stable configuration composed of a piezoelectric beam and a rectangular plate. At low wind speeds, the system exhibits bi-stability, whereas, at high wind speeds, the system exhibits a dynamic tri-stability, which is beneficial for harvesting variable-speed wind energy. The theoretical analysis was carried out. For validation, the prototype was fabricated, and a piezoelectric material was bonded to the beam. The corresponding experiment was conducted, with the wind speed increasing from 1.5 to 7.5 m/s. The experiment results prove that the proposed harvester could generate a large output over the speed range. The dynamic stability is helpful to maintain snap-through motion for variable-speed wind. In particular, the snap-through motion could reach coherence resonance in a range of wind speed. Thus, the system could keep large output in the environment of variable-speed wind.

## 1. Introduction

With the development of wireless sensor networks (WSNs), the problem of sensor power is becoming more and more prominent. Thus, harvesting energy from ambient nature and storing it for sensor consumption has received extensive attention in recent decades. In the future, this promising technology may replace the traditional mode of supplying power for sensors with chemical batteries in many extreme environments.

Wind, as a clean, abundant and renewable energy source, is regarded as one of the most promising alternatives to traditional energy. Wind energy harvesting has been studied and developed in both microscale and macroscale [1]. Harvesting wind energy by aerodynamic instability has received increasing attention due to its high power density and easy implementation in practice [2]. Some kinds of wind harvesters have been proposed by exploiting flow-induced vibrations, e.g., flutter, vortex-induced vibration (VIV) and galloping [3,4].

Flutter is a typical flow-induced instability which results from dynamic fluid-structure interaction [5]. According to the mechanism of flutter, the energy harvesters by flutter could be divided into two categories: the extraneously induced energy harvester (EIEH) and the movement-induced energy harvester (MIEH). The flutter motion of EIEH is excited and sustained by a gradient flow pressure caused by von Kárman vortices. One of the primary studies of EIEH was conducted by Allen and Smits [6]. It was demonstrated that the vortex shedding behind a flat plate could be used to excite a piezoelectric membrane to generate electrical power. Akaydin et al. [7,8] placed the flexible piezoelectric cantilever beams in the wake of a circular cylinder to scavenge flow energy. Goushcha et al. [9] immersed a piezoelectric beam in the wake of bluff bodies and employed interaction between the vortex and the flexible beam to harvest flow energy. Hobbs and Hu [10] presented a new piezoelectric harvester that applied tree-like swaying to harvest wind energy. By adjusting the center-to-center distance of the cylinder, the optimal configuration could be obtained. Lahooti and Kim [11] investigated the multi-body interactions in a hydrofoil and its influence on the power generation efficiency. Movement-induced excitation (MIE) is another type of flutter which results from resonantly bending instability caused by the interplay of fluid and elastic forces. Some harvesters were designed based on this mechanism (MIEH). The instability may result in a self-excited motion when the wind speed exceeds the threshold. Tang et al. [12] presented the concept of a flutter-mill based harvester and compared its performance with a representative Horizontal Axis Wind Turbine. Dunnmon et al. [13] put the piezoelectric laminates onto a flexible beam, which could achieve an excellent performance by executing a nonlinear limit cycle motion. The experimental results agree well with the theoretical predictions. The output power per unit length could reach up to 870 W/m at the flow speed of 27 m/s. The response of flexible beam fluttering in viscous flow was studied by Akcabay [14]. The results show that the performance of the harvester depends on the viscous force involved. Moreover, the flutter of a flexible flag was utilized to convert flow energy to electricity [15]. For self-sustained oscillations, the high fluid load could improve the efficiency of energy harvesting. Giacomello et al. [16] explored the flutter instability of a heavy flag hosted ionic-polymer-metal composite. They optimized the parameters of the system so as to maximize the output power. Li et al. [17] proposed a cross-flow stalk-leaf wind energy harvester and tried increasing output power through fluttering motion. The influences of parameters on a harvester from aeroelastic flutter vibration were studied by Bryant [18]. It was found that modifying the parameters, e.g., the flap mass location, the flap mass moment of inertia or the flap mass, could change the cut-in speed and obtain the maximum power. Li et al. [19] compared three operation modes of the polymer piezoelectric energy harvesters and found an optimum value. Zhao et al. [20] designed and experimentally tested three rectangular wings with different aspect ratios. Aquino et al. [21] proposed a Wind-Induced Flutter Energy Harvester (WIFEH). The experimental investigation of the WIFEH was carried out in a wind tunnel. The results examined the WIFEH under various wind tunnel wind speeds varying from 2.3 up to 10 m/s. The WIFEH could generate a RMS voltage of 3 V at *v* = 2.3 m/s. At *v* = 5 m/s, the RMS voltage could reach 4.88 V. Shan et al. [22] presented a novel flutter-induced vibration energy harvester with a curved panel for harvesting energy. The experimental results show that the harvesting performance with the segmented piezoelectric patches is better than that of the continuous ones. A sustained output power density of 0.032 mW/cm^3^ is obtained at the airflow velocity of 25 m/s. Orrego et al. [23] reported the flutter of an inverted piezoelectric flag fixed in an orientation in ambient wind conditions. Generally, the wind energy harvesters based on linear mechanism may exhibit a good performance for a flow speed, but will be inefficient if the wind speed fluctuates. On the other hand, the aero-dynamic instability often gives rise to a divergent response, which is not desired for keeping a stable electrical output [24].

To overcome this drawback, some researchers tried introducing nonlinear forces and multi-stability so as to enhance the power output performance [25]. Some investigations focused on multi-stability and its nonlinear characteristics. The results suggest that the multi-stable nonlinear elements are helpful to improve harvesting performance of flow energy. Alhadidi and Daqaq [26] designed a bi-stable wake-galloping energy harvester to improve the response bandwidth for varying wind speed. Zhang et al. [27] used two small magnets to form a bi-stable VIV harvester. Huynh and Tjahjowidodo [28] introduced bi-stable springs to broaden the working range of VIV energy harvester. Nasser et al. [29] performed a comparative study on mono-stable and bi-stable VIV harvesters. The results show that the mono-stable harvester exhibits a hardening behavior, while the bi-stable one exhibits a softening behavior. It is found that both harvesters can widen the synchronization region. Valipour et al. [30] considered a hollow cylindrical tube with flowing fluid, which is supported on a Pasternak-type elastic medium. The parameterized perturbation method was used to solve the nonlinear dynamical equation. The results show that the nonlinear flow-induced frequency will change greatly when the amplitude, flow velocity and nonlocal parameter are large. Inspired by the concept of nonlinear multi-stability, a dynamic-stable flutter energy harvester (DFEH) was proposed. It exhibits different types of stability for different wind speeds. The experimental results proved that it could exhibit bi-stability for the low wind speed and tri-stability for the high wind speed [31]. However, the underlying mechanism was not elucidated fully. In this paper, we carried out the theoretical analysis on the DFEH, and proved that with the increase in flow speed, the system could exhibit tri-stability; then, with the wind speed varying from *v* = 1.5 to 7.5 m/s, we carried out corresponding experimental research; the results proved the transition from the bi-stability to the dynamic tri-stability.

In the natural environment, the wind usually is weak and its speed is variable with time. For WSNs, the node sensors are generally scattered in a large area, and the traditional wind energy harvesting and transmission mode cannot achieve the goal of supplying power to each sensor. Therefore, it is of great significance to design a micro wind energy harvesting structure which could integrate with the sensor to construct a system in the environment of weak and variable-speed wind. The proposed multi-stable harvester could realize snap-through motion, even coherence resonance, over a wide range of wind speeds, thus it could keep a large output for variable-speed wind. The experiment results prove this superiority.

The remainder of the paper is organized as follows: the dynamical model of the DFEH is established first; then, the potential energy analysis is carried out; subsequently, the experiment studies are described for the wind speed ranging from 1.5 to 7.5 m/s; the strain response and the output voltage are obtained for each wind speed, some are magnified to clearly show the jumping characteristics in the bi-stable and dynamic tri-stable states. Finally, some useful conclusions are drawn.

## 2. Dynamic-Stable Flutter Energy Harvester

**Figure 1:**
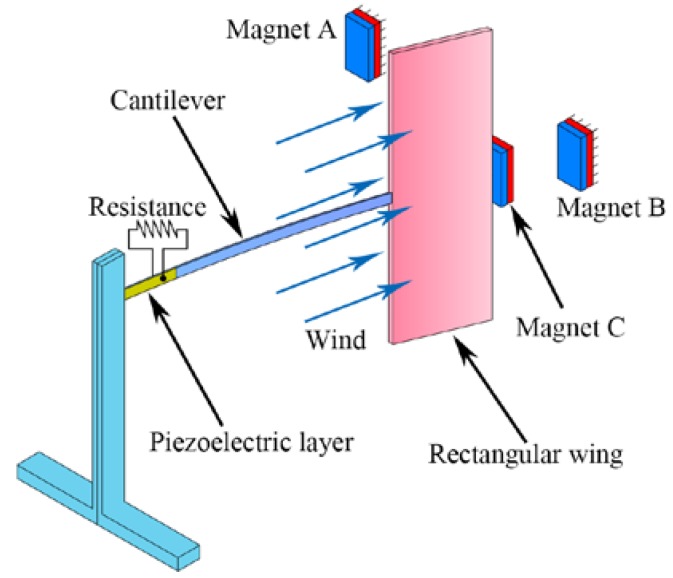
Schematic of the proposed dynamic-stable flutter energy harvester (DFEH).

The schematic of the proposed DFEH is illustrated in Figure 1, which consists of a piezoelectric cantilever, a rectangular wing and three magnets. The tip and fixed magnets are designed to be attractive, in such a way that the piezoelectric beam can possess a bi-stable characteristic. The rectangular wing is designed such as to induce flutter instability in the air field, which may lead the system to approach one static equilibrium position or jump between two equilibrium positions. Furthermore, as the incoming flow passes through, the combined effect of elastic force, magnetic force and aerodynamic force could make the system have different equilibrium positions for different wind speeds. This characteristic is helpful for exciting snap-through motion for variable-speed wind.

The dynamical characteristics of the DFEH may be reflected by its potential energy feature. Therefore, for the first step, we derived its potential energy. The total potential energy of the DFEH includes two parts, i.e, the elastic part and the magnetic part.

The elastic potential energy can be calculated from Euler–Bernoulli theory and Hooke’s law. For an elastic beam, the strain and stress can be given by

(1) $$\varepsilon_{xx}=-z\frac{\partial w\left(x,t\right)}{\partial x^{2}}$$

(2) $$\sigma_{xx}=E_{s}\varepsilon_{xx}$$

where $\varepsilon_{xx}$ and $\sigma_{xx}$ represent the normal stress and the normal strain, respectively; $E_{s}$ is the Young’s modulus of the beam; *w*(*x*,*t*) represents the transverse displacement at distance x from the clamped side and at instant t. Then, the elastic potential energy of the beam can be given by:

(3) $$U_{s}=\frac{1}{2}\int_{0}^{L_{s}}\int_{A}\sigma_{xx}\varepsilon_{xx}dAdx$$

where *A* is the area of cross section of the beam. Substituting Equations (1) and (2) into Equation (3) yields

(4) $$U_{s}=\frac{1}{2}E_{s}I_{s}\int_{0}^{L_{s}}{\left[w^{\prime \prime }\left(x,t\right)\right]}^{2}dx$$

where $I_{s}$ is the inertia moment and can be formulated as $I_{s}=\frac{1}{12}b_{s}h_{s}^{3}$.

Similarly, the elastic potential energy of piezoelectric laminate can be given by

(5) $$U_{p}=\frac{1}{2}E_{p}I_{p}\int_{0}^{L_{p}}{\left[w^{\prime \prime }\left(x,t\right)\right]}^{2}dx$$

where $I_{p}$ is the inertia moment of piezoelectric laminate and can be given by $\frac{1}{12}b_{p}h_{p}\left(4h_{p}^{2}+6b_{p}h_{s}+3h_{s}^{2}\right)$; $E_{p}$ is the Young’s modulus of piezoelectric laminate.

Moreover, the elastic potential energy of rectangular wing can be given by

(6) $$U_{Fp}=\frac{1}{2}E_{Fp}I_{Fp}\int_{L_{s}-L_{Fp}}^{L_{s}}{\left[w^{\prime \prime }\left(x,t\right)\right]}^{2}dx$$

where the $E_{Fp}$ is Young’s modulus of rectangular wing; $I_{Fp}$ is the inertia moment of the rectangular wing.

As a result, the total elastic potential energy of system can be given by

(7) $$\begin{array}{c} U_{e}=U_{s}+U_{p}+U_{Fp}\\ =\frac{1}{2}E_{s}I_{s}\int_{0}^{L_{s}}{\left[w^{\prime \prime }\left(x,t\right)\right]}^{2}dx+\frac{1}{2}E_{p}I_{p}\int_{0}^{L_{p}}{\left[w^{\prime \prime }\left(x,t\right)\right]}^{2}dx\\ +\frac{1}{2}E_{Fp}I_{Fp}\int_{L_{s}-L_{Fp}}^{L_{s}}{\left[w^{\prime \prime }\left(x,t\right)\right]}^{2}dx \end{array}$$

**Figure 2:**
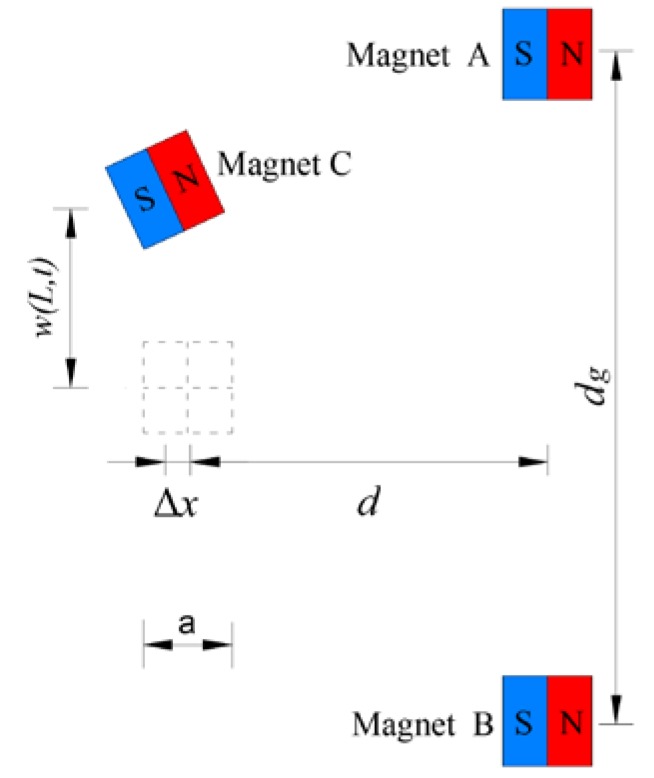
Geometric configuration of the tip magnet and two external magnets.

In deriving magnetic potential energy, the three magnets could be modeled as point dipoles. The geometric configuration of the tip magnet and two external magnets is illustrated in Figure 2. The total magnetic potential energy generated by magnets A and B upon magnet C could be given by [32]

(8) $$U_{m}=-\frac{\mu_{0}}{4\pi }\left(\nabla \frac{m_{A}\cdot r_{AC}}{\|r_{AC}{\|}_{2}^{3}}\right)\cdot m_{c}-\frac{\mu_{0}}{4\pi }\left(\nabla \frac{m_{B}\cdot r_{BC}}{\|r_{BC}{\|}_{2}^{3}}\right)\cdot m_{c}$$

where $m_{A}$ ($m_{B}$ or $m_{c}$) denotes the magnetic moment vectors of magnet A (B or C); $\alpha =\arctan\left[w^{\prime }\left(L,t\right)\right]$ is the rotation angle of beam tip; $\mu_{0}$ is the magnetic permeability constant.

Considering the beam’s deflection and the tip magnet’s size, the vertical displacement of magnet C can be represented by $w\left(L,t\right)+\frac{a\sin\alpha }{2}$, where *a* is the side length of magnet C. Then, vectors $r_{AC}$ and $r_{BC}$, which are directed from the magnetic moment source of magnet A and B to that of magnet C, will become

(9) $$r_{AC}=\left[\begin{array}{cc} d+\Delta x & w\left(L,t\right)+\frac{a}{2}\left(1-\cos\alpha \right)-\frac{1}{2}d_{g} \end{array}\right]$$

(10) $$r_{BC}=\left[\begin{array}{cc} d+\Delta x & w\left(L,t\right)+\frac{a}{2}\left(1-\cos\alpha \right)+\frac{1}{2}d_{g} \end{array}\right]$$

$m_{A}$ ($m_{B}$ or $m_{c}$) can be estimated from magnetization intensity $M_{A}$ ($M_{B}$ or $M_{C}$) and material volume $V_{A}$ ($V_{B}$ or $V_{c}$). They can be expressed by the following formulas:

(11) $$m_{A}=\left[\begin{array}{cc} 0 & -M_{A}V_{A} \end{array}\right]$$

(12) $$m_{B}=\left[\begin{array}{cc} 0 & -M_{B}V_{B} \end{array}\right]$$

(13) $$m_{C}=\left[\begin{array}{cc} M_{C}V_{C}\cos\alpha & M_{C}V_{C}\sin\alpha \end{array}\right]$$

It should be noted that the horizontal displacement of magnet A is $\Delta x=\frac{a}{2}\left(1-cos\alpha \right)$. Since α≈0, then Δx≈0. As a result, the magnetic potential energy expression can be written as

(14) $$\begin{array}{c} U_{m}=\frac{\mu_{0}M_{A}M_{C}V_{A}V_{C}\left(-{\left(w\left(L,t\right)+\frac{1}{2}d_{g}\right)}^{2}+2d^{2}-3d\left(w\left(L,t\right)+\frac{1}{2}d_{g}\right)w^{\prime }{}^{\left(L,t\right)}\right)}{4\pi \sqrt{{w^{\prime }{}^{\left(L,t\right)}}^{2}+1}{\left({\left(w\left(L,t\right)+\frac{1}{2}d_{g}\right)}^{2}+d^{2}\right)}^{5/2}}\\ +\frac{\mu_{0}M_{B}M_{C}{V_{BV}}_{C}\left(-{\left(w\left(L,t\right)+\frac{1}{2}d_{g}\right)}^{2}+2d^{2}-3d\left(w\left(L,t\right)+\frac{1}{2}d_{g}\right)w^{\prime }\left(L,t\right)\right)}{4\pi \sqrt{w^{\prime }\left(L,t\right){}^{2}+1}{\left({\left(w\left(L,t\right)+\frac{1}{2}d_{g}\right)}^{2}+d^{2}\right)}^{5/2}} \end{array}$$

**Figure 3:**
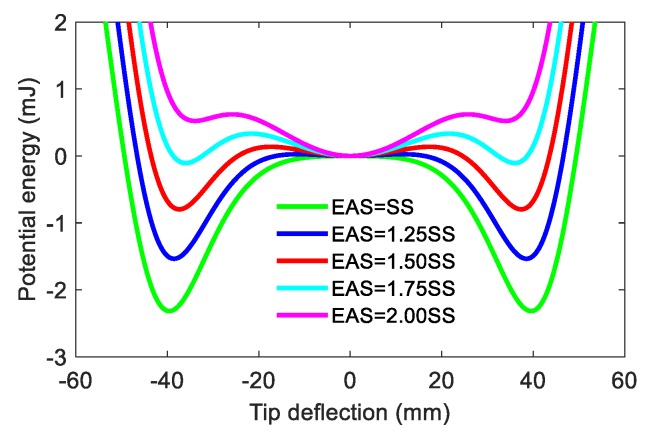
Variations of the potential energy function with tip deflection for different equivalent aero-dynamic stiffness (EAS) values.

The material and geometric parameters in simulation and experiment are the same and are listed in Table 1. It should be noted that the stability of the neutral equilibrium position depends on the wind speed, i.e., it is unstable in the static state and will become stable in high-speed wind. When the wind blows through the DFEH, the aero-dynamical force acting on the rectangular wing will create a disturbance, leading the beam tip to move and then to be attracted by the fixed magnets. At this stable equilibrium position, owing to the deflection of the beam, the effect of aero-dynamical force will change and act as a restoring force, making the beam return to the neutral position. The repeat of this process could produce a large-amplitude vibration and thus generate a large output. To account for the aero-dynamical influence on potential energy, we define an equivalent aero-dynamic stiffness (EAS). The EAS is the force required to make the beam tip keep a unit displacement for a certain wind speed. As is known, the higher the wind speed is, the more force is needed to lift the beam’s tip, i.e., EAS will increase with the wind speed. Figure 3 shows the influence of EAS on the potential energy, where SS is the structure stiffness. We can see that with the increase in EAS, the DFEH’s potential well number turns to three from two, i.e., it goes to tri-stability from bi-stability. 

From the potential energy analysis, it can be seen that the DFEH is a bi-stable system when it is subjected to a relatively low-speed incoming flow; then, when the flow speed increases and exceeds a threshold value, the DFEH will exhibit a tri-stable characteristic, whose three potential wells will make the snap-through motion easily occur. Therefore, the dynamic stability enables the DFEH to execute snap-through motion in both low and high wind speeds. Thus, the system could realize snap-through motion, and even coherence resonance in the environment of variable-speed wind.

## 3. Experimental Verification

**Figure 4:**
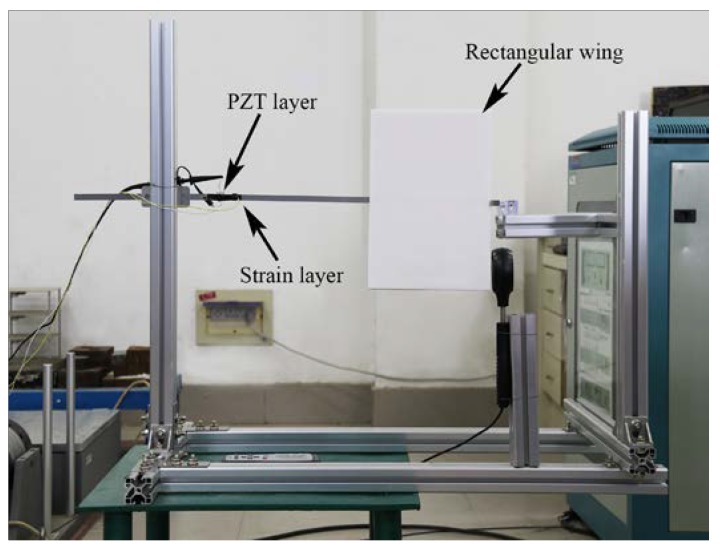
Experimental setup of the DFEH.

**Figure 5:**
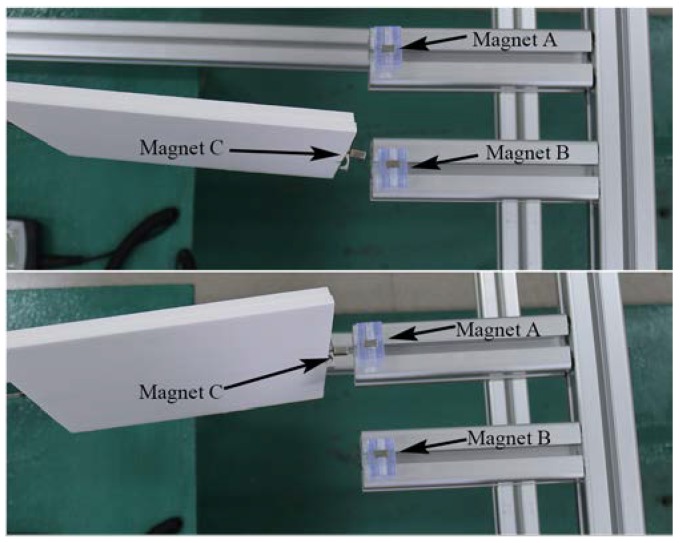
Two static stable positions of the DFEH.

**Figure 6:**
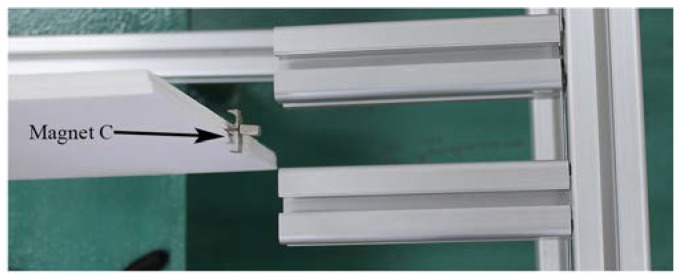
One stable position of the linear flutter energy harvester (LFEH).

The corresponding experiment was carried out to verify the advantages of DFEH. Figure 4 shows the prototype of DFEH and the experimental setup; the DFEH is composed of a cantilever substrate and a rectangular wing. A piezoelectric patch and a strain sensor were bonded to the cantilever. A data acquisition device (DH5922D, DONG HUA) was employed to measure and record the dynamic strain signal and the dynamic piezoelectric voltage across a resistive load (R = 5 MΩ), which were selected as the representative quantities for harvesting performance. The air speed was measured with a digital anemometer (AS8336, XI MA). Figure 5 illustrates the stable equilibrium positions of DFEH. As is clear from Figure 5, the DFEH has two static stable positions, one is near magnet A, the other is near magnet B. If the two fixed magnets are removed, the system degenerates to a linear flutter energy harvester (LFEH), which was put in the same wind field for comparison. The stable equilibrium position of LFEH is shown in Figure 6, which is at the neutral position.

**Figure 7:**
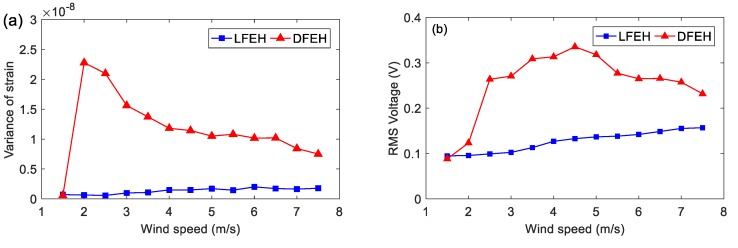
(**a**) Variance of dynamic strain and (**b**) root-mean-square (RMS) voltage versus wind speed (LFEH and DFEH).

Figure 7 shows the variation in dynamic strain variance and root-mean-square (RMS) of voltage with the wind speed, which ranges from 1.5 to 7.5 m/s. It is apparent that the DFEH exhibits a much better performance than the LFEH. Moreover, in Figure 7a, there appears to be a sharp increase in the variance of strain for the DFEH, corresponding to the occurrence of snap-through motion. The snap-through motion could lead to an increase in output voltage. Moreover, the DFEH can maintain the snap-through motion over a wide range of wind speeds, thus keeping a large output. It should be noted that for the wind speeds larger than *v* = 2.0 m/s, the strain variance decreases monotonously with the wind speed, but the resulting voltage increases reversely and even reaches the maximum value at *v* = 4.5 m/s. This is due to the increase in EAS and the emergence of an additional neutral equilibrium position.

In order to show the dynamic-stable characteristics of the DFEH for different wind speeds clearly, the dynamic strain and resulting voltage, in terms of time and spectrum domains, are shown in Figure 8, Figure 9, Figure 10, Figure 11, Figure 12, Figure 13, Figure 14, Figure 15, Figure 16, Figure 17, Figure 18, Figure 19 and Figure 20, with the wind speed varying from 1.5 to 7.5 m/s.

**Figure 8:**
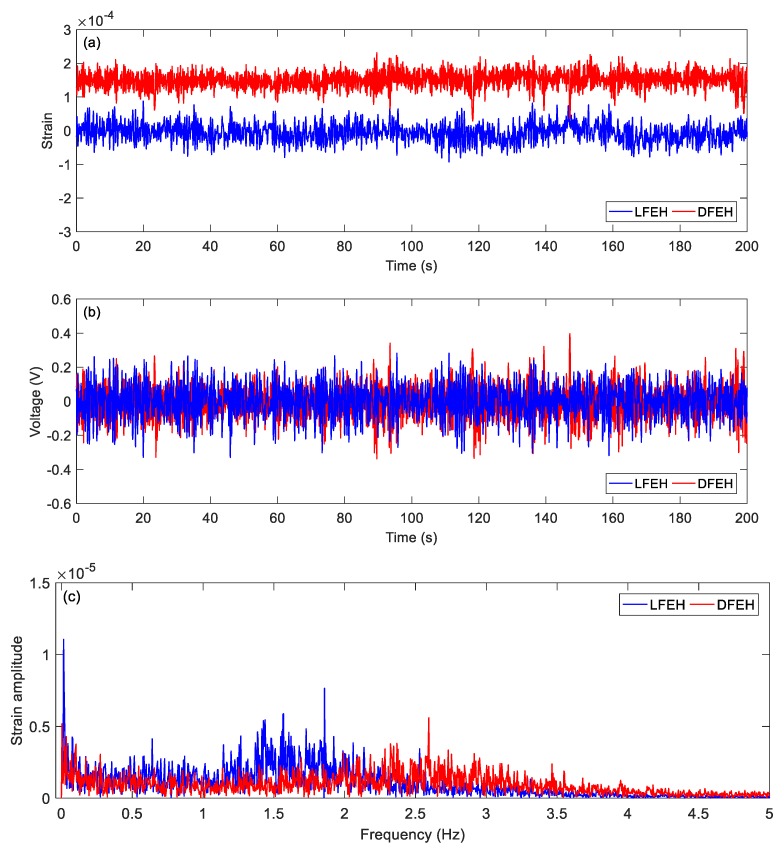
(**a**) Time histories of strain response, (**b**) time histories of voltage response, (**c**) spectrum of dynamic strain response (v = 1.5 m/s).

First, at *v* = 1.5 m/s, i.e., a very low speed, the DFEH behaves like a linear one. Its strain and voltage are shown in Figure 8a,b. It can be seen that both DFEH and LFEH oscillate in one potential energy well now. The corresponding spectra of strain responses are shown in Figure 8c.

**Figure 9:**
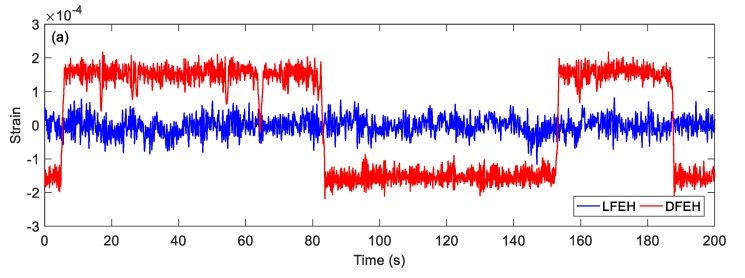

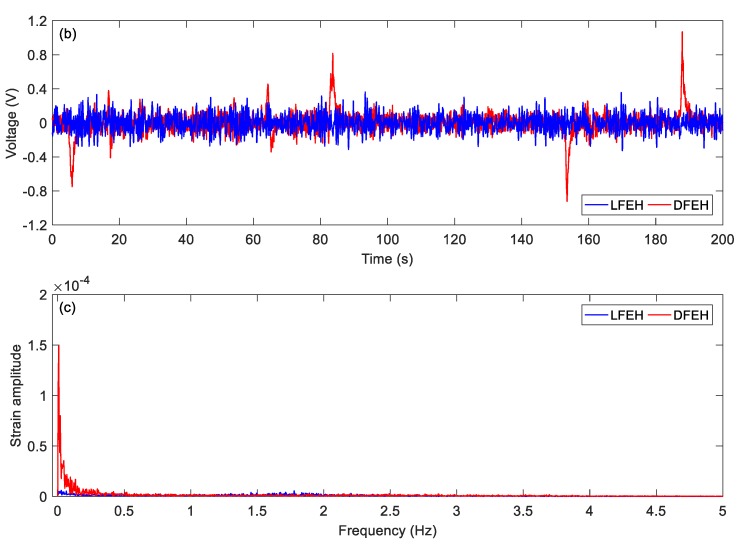
(**a**) Time histories of strain response, (**b**) time histories of voltage response, (**c**) spectrum of dynamic strain response (*v* = 2.0 m/s).

Then, increasing the wind speed to *v* = 2.0 m/s, the DFEH’s dynamic strain shows that the DFEH begins to jump between two static equilibrium positions (Figure 9a). The snap-through motion results in a large amplitude and a high output voltage (as shown in Figure 9b). The spectra of strain show that there exists a large component in the low frequency region.

**Figure 10:**
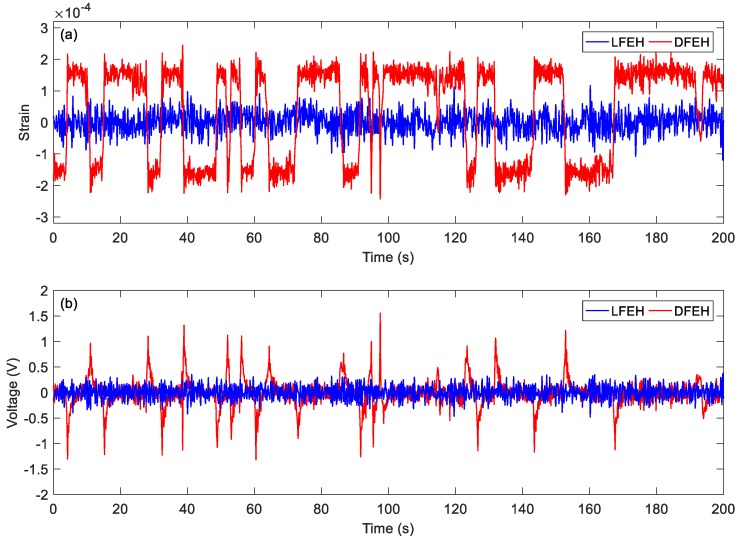

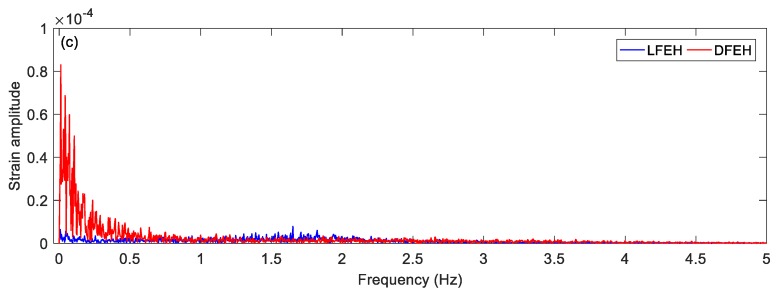
(**a**) Time histories of strain response, (**b**) time histories of voltage response (**c**) spectrum of strain (*v* = 2.5 m/s).

At v = 2.5 m/s, jumping happens more frequently. The jumping motion results in high pulses in the output voltage, as shown in Figure 10a,b.

**Figure 11:**
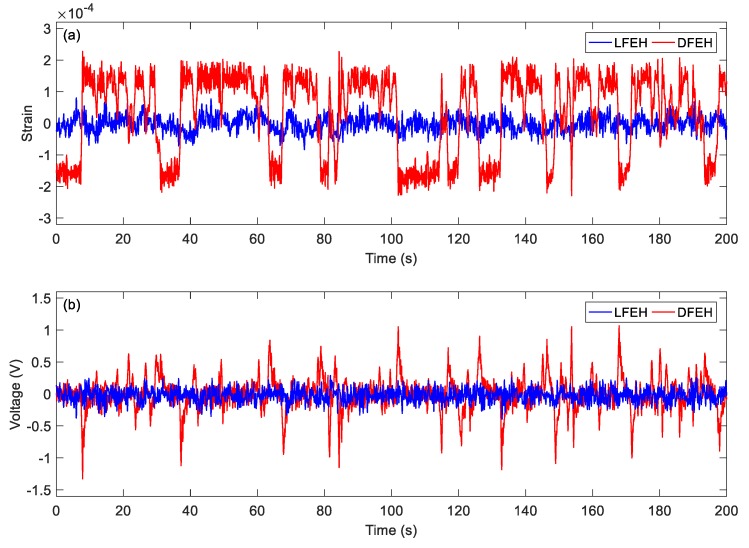
(**a**) Time histories of strain response, (**b**) time histories of voltage response (*v* = 3.0 m/s).

When the wind speed reaches *v* = 3.0 m/s, it can be found that an additional equilibrium position emerges. In this case, the jumping motion could happen between any two of three equilibrium positions, as shown in Figure 11a. This phenomenon can be maintained till *v* = 7.5 m/s.

Then, at *v* = 3.5 m/s, the results show that two types of jumping could take place simultaneously. Although now jumping between the adjacent equilibrium positions produces relatively small pulse voltages, it can happen more frequently. Therefore, the output voltage is still quite large.

As the wind speed increases to *v* = 4.0 m/s, the jumping happens mostly between the static and the neutral equilibrium positions; since it happens densely, the output voltage increases accordingly.

**Figure 14:**
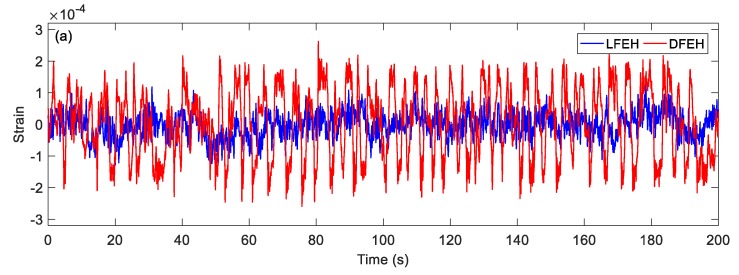

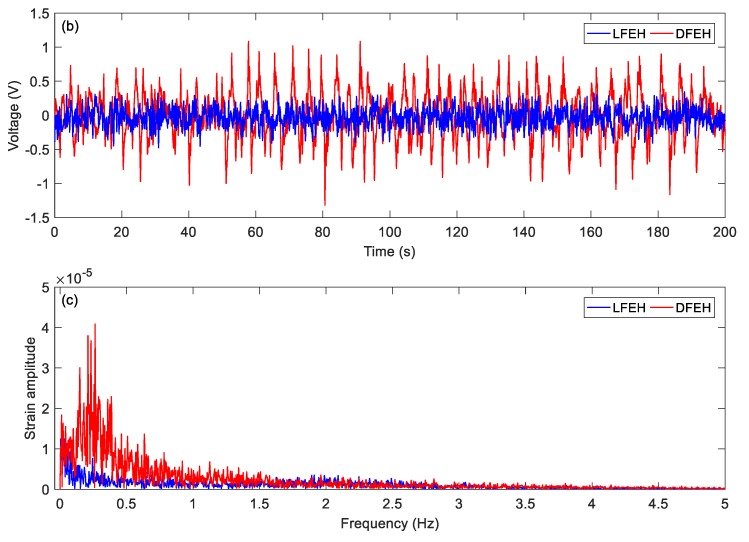
(**a**) Time histories of strain response, (**b**) time histories of voltage response, (**c**) spectrum of dynamic strain response (*v* = 4.5 m/s).

When the wind speed reaches *v* = 4.5 m/s, it follows from Figure 14 that the jumping could happen densely between all three equilibrium positions. Accordingly, the corresponding output voltage increases greatly. From the spectrum, it can be observed that now there appears a peak in the low frequency region, indicating that the jumping exhibits a nearly periodic feature, i.e., the DFEH attains coherence resonance now.

**Figure 15:**
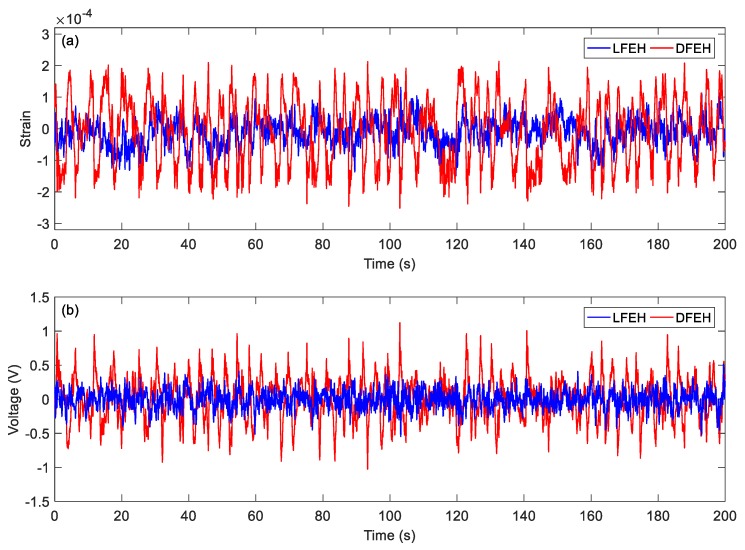

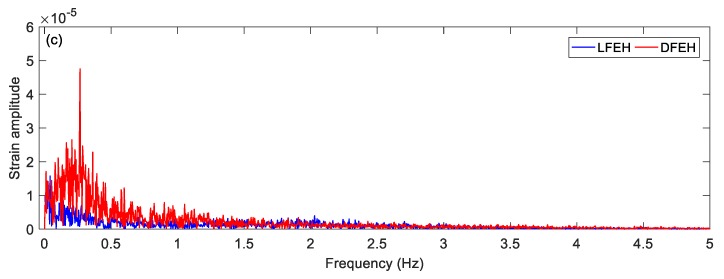
(**a**) Time histories of strain response, (**b**) time histories of voltage response, (**c**) spectrum of dynamic strain response (*v* = 5.0 m/s).

At *v* = 5.0 m/s, as shown in Figure 15, the jumping between potential wells remains dense, thereby producing dense pulses in the output voltage. The corresponding spectra show that the DFEH has a dominant nearly periodic component, suggesting that the coherence resonance is happening.

**Figure 16:**
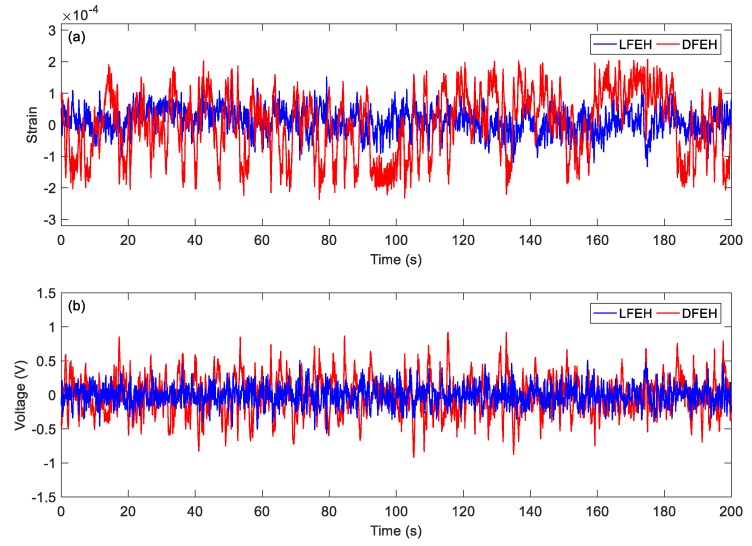
(**a**) Time histories of strain response, (**b**) time histories of voltage response (*v*=5.5 m/s).

**Figure 17:**
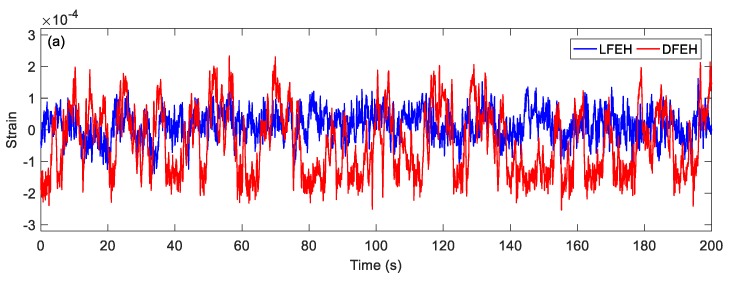

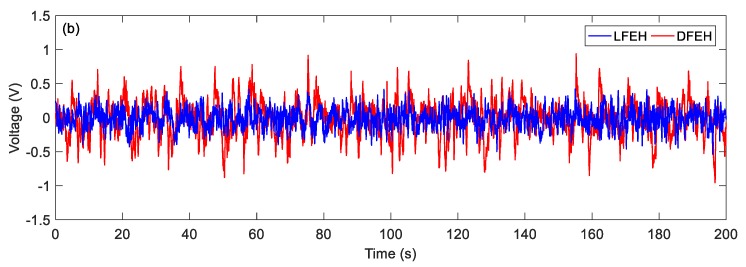
(**a**) Time histories of strain response, (**b**) time histories of voltage response (*v* = 6.0 m/s).

**Figure 18:**
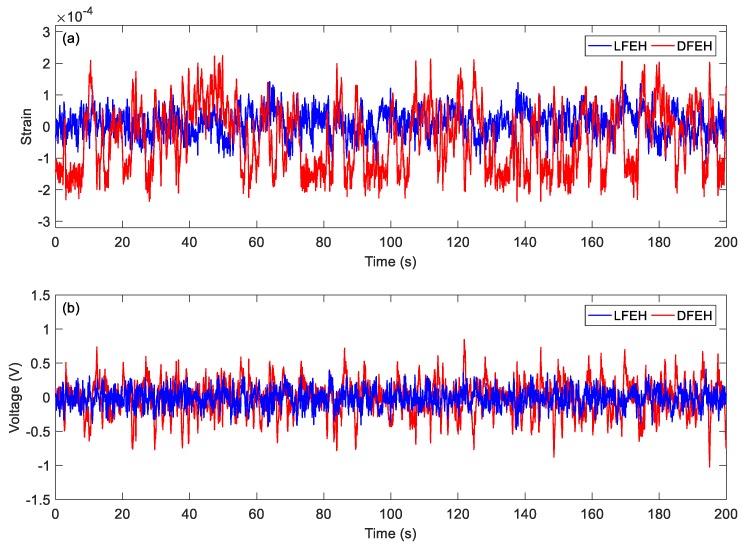
(**a**) Time histories of strain response, (**b**) time histories of voltage response (*v* = 6.5 m/s).

**Figure 19:**
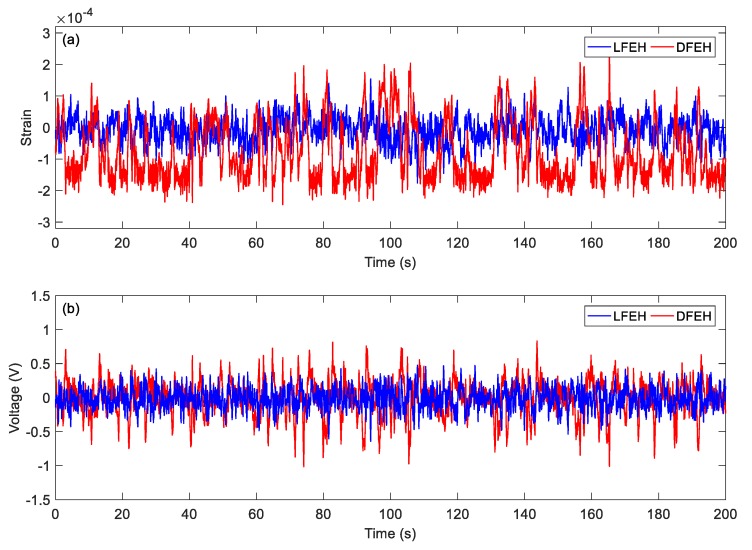
(**a**) Time histories of strain response, (**b**) time histories of voltage response (*v* = 7.0 m/s).

**Figure 20:**
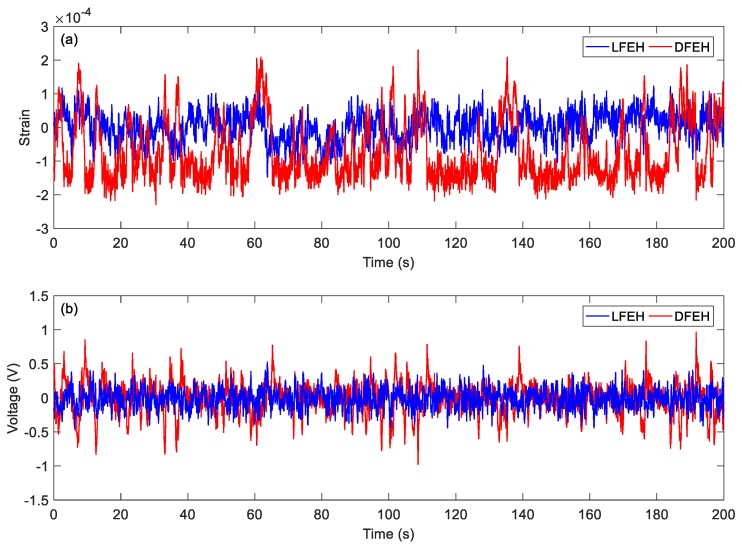
(**a**) Time histories of strain response, (**b**) time histories of voltage response (*v* = 7.5 m/s).

We increased the wind speed to *v* = 7.5 m/s. The corresponding results are shown in Figure 16, Figure 17, Figure 18, Figure 19 and Figure 20. Since the wind speed is relatively high now, the emerged neutral equilibrium position becomes more stable, at which the response stays for more time. Thus, the jumping between the adjacent equilibrium positions becomes dominant. However, in this case, the output voltage is still quite large, since the adjacent jumping is easier to occur and the times of jumping increase.

**Figure 21:**
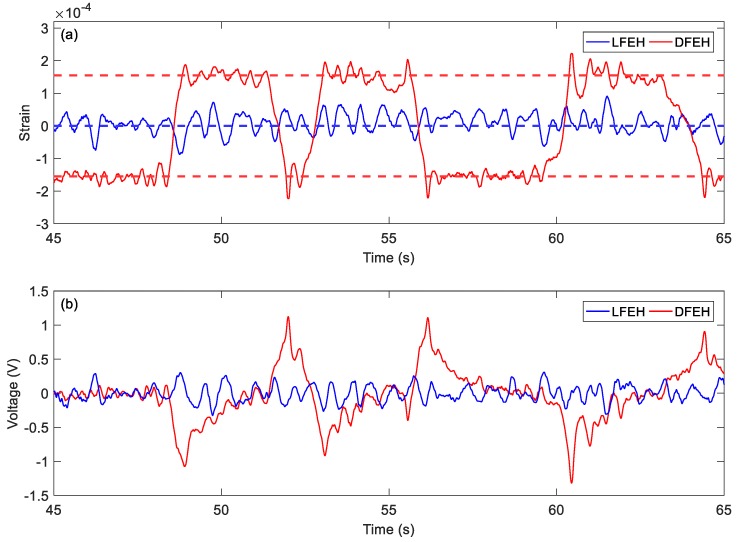
Bi-stable jumping (**a**) strain response and (**b**) voltage response (v = 2.5 m/s).

**Figure 22:**
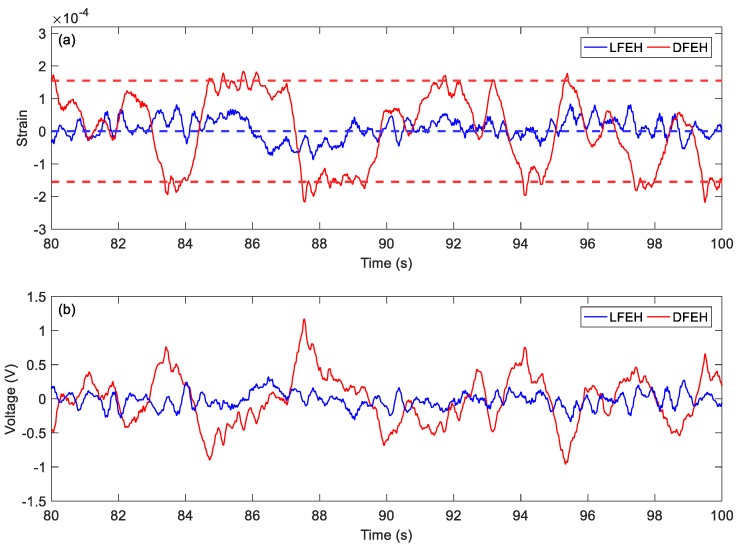
Tri-stable jumping (**a**) strain response and (**b**) voltage response (v = 4.5 m/s).

In order to show the jumping characteristics of the DFEH clearly, for two wind speeds, i.e., *v* = 2.5 m/s and *v* = 4.5 m/s, we magnify parts of their strain and voltage responses and plot them in Figure 21 and Figure 22. As shown in Figure 21, at *v* = 2.5 m/s, the system exhibits the bi-stable characteristics, its response jumps between two equilibrium positions; in contrast, at *v* = 4.5 m/s, an additional equilibrium position emerges and the system becomes a tri-stable one, its response jumps between all three equilibrium positions. As for the output voltages, it is apparent that the jumping leads to a spike in the voltage response, thus the dense jumping could generate dense pulses in output voltage and promote the harvesting efficiency.

## 4. Conclusions

In this paper, a dynamic multi-stable flutter harvester is proposed to harvest the energy of variable-speed wind. The validation experiment was carried out. The experimental results prove that it could execute snap-through motion and generated high pulse voltages in the environment of variable-speed wind. At low wind speeds, the harvester jumps between two static equilibrium positions; whereas, at high wind speeds, with a neutral equilibrium position emerging, the harvester could jump between three equilibrium positions. Moreover, the harvester response can reach coherence resonance in a certain range of wind speeds. We believe that this harvester could be directly applied to the actual environment after improvement, due to its simple structure and cheap material. In practice, the size of the structure can be scaled down to adapt to the environment without affecting its performance. Of course, the storage circuit for electric energy needs further design and research, and the connections between the components should be improved.

## Figures and Tables

**Figure 12:**
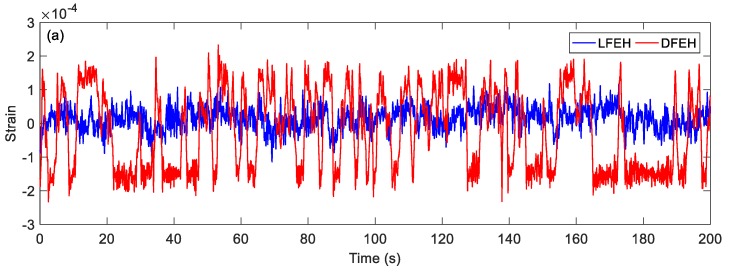

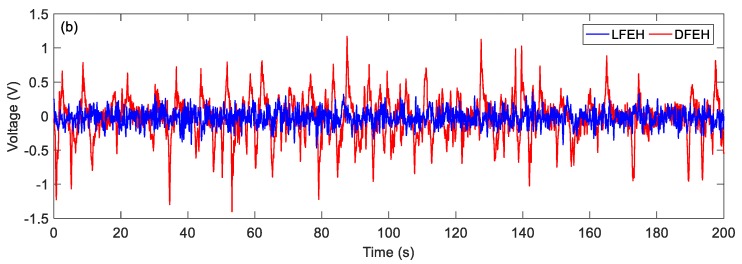
(**a**) Time histories of strain response, (**b**) time histories of voltage response (*v* = 3.5 m/s).

**Figure 13:**
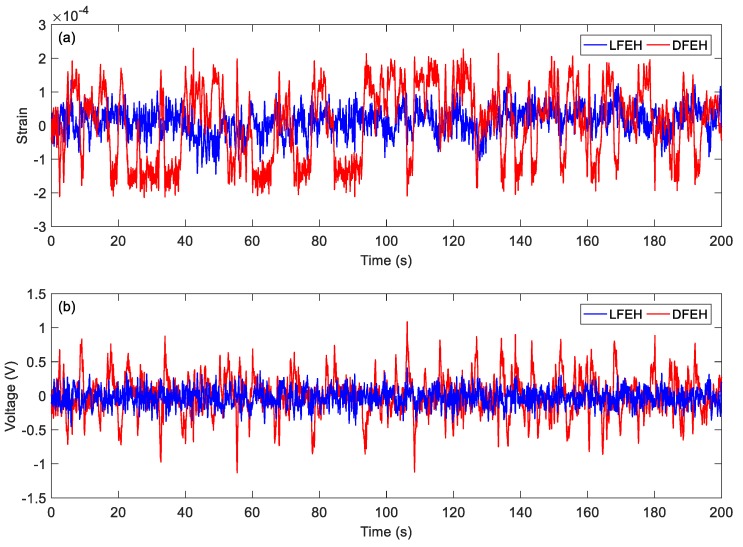
(**a**) Time histories of strain response, (**b**) time histories of voltage response (*v* = 4.0 m/s).

**Table 1:** Model parameters used for simulation and experiment.

Symbol	Description	Value
*Substrate*		
*L_s_*	Length	500 mm
*b*_s_	Width	10 mm
*h*_s_	Thickness	1 mm
*ρ*_s_	Density	7800 kg/m^3^
*E*_s_	Young’s modulus	205 Gpa
*Rectangular wing*		
*L_Fp_*	Length	200 mm
*b_Fp_*	Width	300 mm
*h_Fp_*	Thickness	5 mm
*ρ_Fp_*	Density	18 kg/m^3^
*Piezoelectric material* (PZT-5H, Baoding Hongsheng Ltd.)		
*L*_p_	Length	130 mm
*b*_p_	Width	130 mm
*h*_p_	Thickness	0.15 mm
*ρ*_p_	Density	1785 kg/m^3^
*E*_p_ $\left(c_{xx}^{E}\right)$	Young’s modulus	2 Gpa
*e*_zx_	Piezoelectric coupling coefficient	23 × 10^−10^ C/N
$\epsilon_{zz}^{S}$	Permittivity constant	1.06 × 10^−10^ F/m
*Magnets*		
$M_{A}$, $M_{B}$	Magnetization intensity	1.25 × 10^5^ Am^−1^
$V_{A}$, $V_{B}$	Volume	5 × 10^−7^ m^3^
*μ*_0_	Permeability constant	4π × 10^−7^ NA^−2^
